# Signaling Pathways Regulating Dimorphism in Medically Relevant Fungal Species

**DOI:** 10.3390/pathogens14040350

**Published:** 2025-04-04

**Authors:** Uriel Ramírez-Sotelo, Manuela Gómez-Gaviria, Héctor M. Mora-Montes

**Affiliations:** Departamento de Biología, División de Ciencias Naturales y Exactas, Campus Guanajuato, Universidad de Guanajuato, Noria Alta s/n, col. Noria Alta, Guanajuato C.P. 36050, Mexico; u.ramirezsotelo@ugto.mx (U.R.-S.); m.gomezgaviria@ugto.mx (M.G.-G.)

**Keywords:** transcription factor, environmental stimuli, yeast, hypha, kinase, antifungal drug

## Abstract

Pathogenic fungi that exhibit the ability to alternate between hyphal and yeast morphology in response to environmental stimuli are considered dimorphic. Under saprobic conditions, some fungi exist as filamentous hyphae, producing conidia. When conidia are inhaled by mammals or traumatically inoculated, body temperature (37 °C) triggers dimorphism into yeast cells. This shift promotes fungal dissemination and immune evasion. Some fungal pathogens undergo dimorphism in the contrary way, forming pseudohyphae and hyphae within the host. While temperature is a major driver of dimorphism, other factors, including CO_2_ concentration, pH, nitrogen sources, and quorum-sensing molecules, also contribute to morphological shifts. This morphological transition is associated with increased expression of virulence factors that aid in adhesion, colonization, and immune evasion. *Candida albicans* is a fungus that is commonly found as a commensal on human mucous membranes but has the potential to be an opportunistic fungal pathogen of immunocompromised patients. *C. albicans* exhibits a dimorphic change from the yeast form to the hyphal form when it becomes established as a pathogen. In contrast, *Histoplasma capsulatum* is an environmental dimorphic fungus where human infection begins when conidia or hyphal fragments of the fungus are inhaled into the alveoli, where the dimorphic change to yeast occurs, this being the morphology associated with its pathogenic phase. This review examines the main signaling pathways that have been mostly related to fungal dimorphism, using as a basis the information available in the literature on *H. capsulatum* and *C. albicans* because these fungi have been widely studied for the morphological transition from hypha to yeast and from yeast to hypha, respectively. In addition, we have included the reported findings of these signaling pathways associated with the dimorphism of other pathogenic fungi, such as *Paracoccidioides brasiliensis*, *Sporothrix schenckii*, *Cryptococcus neoformans*, and *Blastomyces dermatitis*. Understanding these pathways is essential for advancing therapeutic approaches against systemic fungal infections.

## 1. Introduction

The ability of pathogenic fungi to change their morphology during their life cycle is widespread; however, few fungi are considered dimorphic, since dimorphism is considered the ability to alternate between two morphologies, yeast and hypha [1]. The changes between these two forms, hypha and yeast, are critical for the pathogenesis, virulence, stress adaptation, and life cycle of dimorphic fungi, and some pathogenic species, known as thermodimorphic fungi, can shift their morphology based on temperature changes. Worldwide, thermodimorphic fungi are responsible for millions of human infections annually [2,3]. Among the best-known medically relevant fungal species with the ability to undergo dimorphism are *Histoplasma capsulatum*, *Paracoccidioides brasiliensis*, *Sporothrix schenckii*, *Candida albicans*, *Cryptococcus neoformans*, and *Blastomyces dermatitis*. These species are responsible for potentially fatal systemic mycoses in humans, particularly in immunocompromised individuals, although healthy individuals are also susceptible to these mycoses [4,5].

The reversible morphological transition between hypha and yeast, known as phase transition, is one of the most important features in the biology and lifestyle of dimorphic fungi. For those species naturally growing on soil or vegetable debris, at a temperature range between 22 and 25 °C, they usually grow as septate hyphae that produce conidia. Soil disturbance by different types of activities can aerosolize conidia and hyphal fragments [1,3]. This process can permit the inhalation of conidia and thus reach different hosts, such as mammals, which have an internal body temperature of 37 °C, which favors the dissemination of the pathogen and the change in its morphology [1]. However, there are exceptions to this rule, since not all pathogenic fungi are saprophytes, and others that are classified as opportunistic infect immunocompromised patients, such as species of the genera *Aspergillus*, *Fusarium*, *Scedosporium*, and *Candida* [6]. Although temperature is the predominant stimulus that influences the phase transition, other stimuli favor these changes, such as the availability of CO_2_, pH, exogenous cysteine, estradiol, nitrogen sources, lipoxygenases, cyclooxygenases, and quorum-sensing molecules [1,7,8].

Fungal dimorphism also has important implications for pathogenesis and immune evasion. For those species forming yeast cells within the host, this morphology favors the dispersion inside the host and also has characteristics that favor infection, such as the ability to survive in macrophages, immune cells that normally destroy pathogens [1,9,10]. In a similar line, those species that grow as hypha within the host have advantages in changing to this morphology, as these cells result big enough to be phagocytosed, or the immune cells may be pierced by hypha growing inside the phagolysosome [11,12]. During morphological change, dimorphic fungi may undergo changes in the cell wall that lead to the reorganization of components, thus preventing the recognition of pathogen-associated molecular patterns by host immune cells [13,14,15,16,17]. Furthermore, morphological change is also associated with the upregulation of specific virulence factors that promote adhesion to host tissues and growth.

In this review, we focused on analyzing the main signaling pathways that govern fungal dimorphism in the transition of mycelial saprobic fungi that change to yeast-like morphology, taking *H. capsulatum* as a model, as well as those fungi that transition oppositely, that is, from yeast-like morphology to the formation of true hyphae, considering *C. albicans* as a biological model. It is relevant to note that *C. albicans* is a polymorphic species that switches between yeast, pseudohyphal, and true hyphal forms, facilitating tissue invasion and immune evasion [15]. Additionally, the white–opaque–grey phenotypic switching, chlamydoconidia formation, and gastrointestinally induced transition (GUT) cell types play a crucial role in mating and adaptation to host environments [18]. Here, we will focus only on the yeast-to-hypha transition, though. The study of pathways controlling dimorphism is crucial not only to understand the biology of these organisms but also to improve prevention, diagnosis, and treatment strategies for systemic fungal infections.

## 2. Fungal Dimorphism Regulators

### 2.1. Temperature-Responsive Genes

Thermally dimorphic fungi possess the ability to transit between two distinct morphological states, hypha and yeast. Currently, in all species studied, dimorphism occurs in response to environmental stimuli. Thermoresistant dimorphic pathogenic fungi, such as *H. capsulatum*, grow on decaying organic matter with a filamentous morphology with the ability to produce vegetative conidia. Following inhalation of hyphal fragments or conidia by the host and subsequent growth at mammalian body temperature, *H. capsulatum* undergoes dimorphism to a yeast-like form capable of growing and spreading as a pathogen in mammals [19]. Because thermally dimorphic fungi can prevail in mammals after an acute infection resolves, it is thought that the parasitic form can return to the saprophytic environment after the death of infected animal hosts, facilitating a transition to the filamentous form, which helps maintain a latent infectious reservoir ready to continue this cycle [19]. In thermodimorphic fungi, genes that control growth in the yeast and filamentous phases have been identified and are considered master regulators governing the transitions, such as Ryp (Ryp1, Ryp2, and Ryp3), the histidine kinase Drk1 (dimorphism-regulating kinase 1), and the GATA family transcriptional regulator Sre1/SreB [20,21,22,23]. In contrast, fungal dimorphism can also occur as a morphological transition from a yeast form to a hypha, which is necessary for dissemination, and *C. albicans* is the best fungal representative with this ability, where the yeast morphology favors its dissemination, and hyphae are associated with tissue invasion. *C. albicans* dimorphism stimulation is also influenced by temperature, and the first step for the transition consists of germ tube formation [24]. In this organism, the family of cytoskeletal filament-forming proteins, septins, has been reported to contribute to morphogenesis by forming a scaffold to capture other proteins that promote septum formation, playing relevant roles during hyphal growth and the maintenance of polarized morphology, as well as proper selection of germ tube formation sites [24]. In *C. albicans*, at least seven genes encoding septins have been reported, where *CDC3* and *CDC12* are essential, but *CDC10*, *CDC11*, and *SEP7*, despite not being considered essential genes, contribute to the formation of the septum and coordinate morphogenesis [24,25,26]. A *C. albicans* heterozygous *cdc12*Δ/*CDC12* mutant grew normally at 30 °C but showed an inability to grow at the temperature that induces hyphal formation (37 °C). Although this mutant formed germ tubes, a strongly polarized hyphal morphogenesis was not observed, as the cells acquired a pseudohyphal morphology, suggesting that septins play a special role in maintaining highly polarized growth [24].

The transmembrane mucins are glycoproteins involved in eukaryotic signaling [27]. In fungi, Msb2 is a regulator of environmental stress, cell wall biosynthesis, and the CEK pathway [27]. Msb2 plays a key role in *C. albicans* thermal adaptation, since Msb2 is essential for the hyphal formation and growth at 42 °C, regulating the high-temperature response through the CEK pathway [27]. Ssa1, an Hsp70 chaperone, and abundant cell wall component, was also found to be critical for thermal adaptation and the regulation of the MAP kinase pathways (CEK and PKC) [27].

It is known that in *H. capsulatum*, the transcription factors Ryp1–4 (required for yeast-phase growth) are responsible for regulating the dimorphic change in response to temperature [28]. Through insertional mutagenesis, Nguyen and Sil [20] identified *RYP1*, which is required for *H. capsulatum* growth at 37 °C, and the mutants in this gene grew with filamentous morphology constitutively regardless of temperature. Furthermore, *RYP1* expression analysis revealed that it is required for the regulation of yeast phase-specific genes, as well as the regulation of two key genes in *H. capsulatum* virulence: the virulence factor *CBP1* and *YPS3*, which are required for the colonization of organs, such as the mouse lungs, liver, and spleen [20]. Ryp2 and Ryp3 are two additional regulators in *H. capsulatum* necessary for its growth as yeast at 37 °C [21]. Mutants in *RYP2* and *RYP3* genes also lost their ability to grow as yeast at 37 °C and had defects in conidiation. Moreover, when murine bone marrow-derived macrophages were infected with conidia of the *RYP2* or *RYP3* mutant strains, most of the conidia did not germinate or were nonviable [21]. The fourth transcriptional regulator, RYP, was identified as a Zn(II)^2^Cys6 zinc binuclear cluster domain protein, whose expression analysis revealed that its transcription levels are higher in the yeast phase at 37 °C than in hyphae [29]. The transcription factors Ryp1-3 interact directly with DNA, particularly with a large set of genes related to virulence and cellular morphology [29]. Even though Ryp4 acts synergistically with Ryp1-3, its regulation is independent of them [29]. This temperature-responsive positive regulation circuit governs *H. capsulatum* dimorphism, generating the infective phase [29].

The heat shock proteins (HSPs) play a relevant role in fungal thermodimorphism and pathogenesis because they modulate cell signaling in response to temperature variations associated with the transition from the saprobic stage to the warm environment in the host [30]. In *H. capsulatum*, it has been identified that Hsp70 is involved in the change from hypha to yeast, depending on the temperature change [31]. The *H. capsulatum HSP70* gene shares sequence homology with *HSP70* genes from other dimorphic fungi, such as *Paracoccidioides brasiliensis* [32]. The expression of this gene is also inducible by the change in the *P. brasiliensis* growth temperature, as well as in the transition from hypha to yeast, although its expression is differentially higher in yeast morphology [32]. Surprisingly, when the incubation of *P. brasiliensis* yeast was increased to 42 °C, a downregulation of the expression of several genes encoding other cellular proteins occurred, but there was a higher expression of four genes possibly corresponding to HSPs [32]. This fact suggests that Hsp70 expression is closely related to cell differentiation as a response of *P. brasiliensis* to temperature changes [32]. Other proteins that belong to this family include Hsp90 and have already been described for *H. capsulatum*, *C. neoformans*, *A. fumigatus*, and *P. brasiliensis*, among others. Hsp90, unlike other HSPs, is associated with a specific subset of proteins in the cell, called “client proteins”, promoting their folding and maturation, such that Hsp90 generates protein folding reservoirs that can compensate for the phenotypic impacts of mutations in client proteins, facilitating evolutionary change [33]. *H. capsulatum* Hsp82, an Hsp90 family member, is also heat-inducible and is predominantly expressed at 37 °C once morphogenesis begins in hyphae [34]. Insertion mutants affected in the Hsp82 expression levels showed attenuated virulence when confronted with bone marrow-derived macrophages, even though the growth of this mutant at 37 °C was normal [35,36]. The gene encoding Hsp90 has been identified as a single copy in the *P. brasiliensis* genome, it is upregulated in yeast morphology, and its expression is upregulated in the initial stage of the hypha-to-yeast transition [37]. Furthermore, Hsp90 from *P. brasiliensis* acts synergistically with calcineurin to promote dimorphism and prevents the generation of reactive oxygen species (ROS) by stimulating antioxidant defenses during thermal stress [38].

*C. albicans* Hsp90 governs morphogenesis, such that engagement of the Hsp90 function induces the transition from yeast to hypha in the absence of any additional inducing signal and makes it temperature-dependent by inhibiting signaling through the cAMP/protein kinase A (PKA) pathway [33]. Furthermore, Hsp90 stabilizes other kinases of the cell wall integrity pathway, including Pkc1, Bck1, Mkk2, and Mkc1. Other proteins linked to Hsp90 include the cyclin Pcl1, the cyclin-dependent kinase Pho85, and the transcription factor Hms1, which are required for filamentation in response to Hsp90 inhibition and elevated temperature [33]. On the other hand, the heat shock transcription factor Hsf1 also plays a central role in regulating transcriptional responses following heat stress, including the upregulation of genes encoding other chaperones, such as *HSP70*, *HSP104*, and the same *HSP90*. Thus, the modulation of *HSF1* levels also has a profound impact on morphogenesis [33].

### 2.2. Mating Is Associated with Dimorphism

Sexual reproduction in fungi is directed by a specialized region of the genome known as the mating-type (*MAT*) locus that confers cell type identity and controls fungal development during the sexual cycle [39]. Fungi can have self-compatibility to mating, meaning they do not need a genetically different partner (homothallic), or have one of two idiomorphs to mating (heterothallic) [39]. *C. neoformans* is a heterothallic fungus, and its sexual reproduction is governed by this sex locus *MAT*, containing one of two possible idiomorphs, *MATα* or *MATa* [40], which determine sexual compatibility, that is, one locus encodes homeodomain transcription factors, and the other encodes pheromones and pheromone receptors [41]. Fungal cells with either the *MATa* or *MATα* idiomorph are morphologically indistinguishable, their growth is similar in laboratory cultures, and their mating occurs with the same ease in both cases, obtaining offspring in the same *MATa* and *MATα* proportions [40]. In *C. neoformans*, only strains with the *MATα* idiomorph develop conjugation tubes that elongate and fuse with *MATα* cells during mating. Then, *MATα* cells develop dikaryotic hyphae whose meiotic products give rise to basidiospores, in a similar way as occurs in dioecious animals and plants [40]. The mating types *MATa* and *MATα* in *C. neoformans* have a connection with the global transcriptional repressor *TUP1* involved in the regulation of fungal growth and dimorphism, since the disruption of *TUP1* in a wild-type *MATα* strain crossed with another test strain (JEC32) resulted in extensive mating hyphae after 2 days of incubation; conversely, the hyphal development of the cross between the reintegrating strain and the test strain JEC32 was significantly reduced [40]. The frequency of mating by *TUP1* reintegrants was restored, whereas *tup1* null mutants transformed with the vector were not. These results indicate that loss of *TUP1* reduces mating efficiency, and, overall, *TUP1* has an important effect on the sexual reproduction of *C. neoformans* [40]. Another transcriptional factor associated with mating type and that also regulates the signal transduction pathway of the mitogen-activated protein (MAP) kinase cascade is Ste12p [42]. It is also known that the MAPK pathway is directly involved in pheromone detection; in turn, the main elements of this pathway coordinate morphogenesis in several fungal species, including *C. neoformans* [43]. This transcription factor regulates mating, pseudohyphal development, and haploid invasive growth [44]. A *C. neoformans ste12α* null mutant did not show defects in yeast growth; however, it lost its ability to carry out haploid fruiting in a culture medium that favored filamentation [45]. In addition, the *STE12α* deletion altered the expression of some virulence-associated genes, such as *CNLAC1*, *CAP59*, *CAP60*, and *CAP64*. Consequently, the *C. neoformans ste12*α showed a global reduction in virulence [42,45]. Even genes that are not in the *MAT* locus, such as *CPR2*, *STE7*, and *CPK1*, are also involved in dimorphism and mating [43]. The *C. neoformans* homeodomain proteins Sxi1α or Sxi2a are elements that control newly fused cells, resulting in them being unable to initiate a new developmental pathway, contributing to the specification of the a + α state of the newly formed dikaryon. The absence of these proteins causes fused cells to arrest and be unable to form filaments, basidia, or dikaryotic spores [46].

Ascomycetes belonging to the *Candida* genus, such as the opportunistic pathogen *C. albicans*, were previously considered asexual or imperfect yeasts. However, there is currently evidence demonstrating their capacity for sexual or parasexual reproduction [47]. In the *Candida* genus, there are haploid or diploid species, where the haploids have the potential to carry out complete sexual cycles, such as *Candida lusitaniae*. Despite not having the molecular machinery typically necessary to carry out meiosis, Ste12 seems to be the transcription factor essential for mating and meiosis [48,49,50]. On the other hand, in *C. albicans*, which is representative of the diploid species, *MTL* was identified, and it was shown that mating also involves a and α idiomorphs, where successful mating requires that the cells undergo a phenotypic change from the preestablished white state to the competent opaque state [48]. The *C. albicans* change of the white to opaque state regulates mating to such an extent that it influences other aspects, including filamentation, interaction with the host immune cells, and even virulence [48,51,52,53].

Until now, no work has reported a thorough genetic analysis of the association between mating and signaling pathways that govern dimorphism in *H. capsulatum*.

### 2.3. Signaling Cascades in Dimorphic Fungi

All living organisms share the elementary characteristic of responding to external stimuli. Cells transform almost all external signals from their environment into required cellular responses through the activation of suitable signaling pathways [54]. The evolution of multicellular organisms, together with the specialization of the cells that shape them, has considerably increased the complexity of signaling pathways. These types of cellular responses are associated with basic elements such as growth and proliferation, to more complex processes like the development of structural support elements, transport, and supply of nutrients, among others [54]. When talking of fungal pathogens, the host represents a hostile environment, and signaling pathways help the fungal cell to adapt to these unfavorable conditions, such as body temperature, pH, and the particular host milieu. In addition, for growth, as for any other living organism, pathogenic fungi require essential nutrients for their development, such as carbohydrates, amino acids, nitrogen, and trace elements [55]. In fungi, the pathway for nutrient sensing is closely related to pheromone responsiveness for mating, growth, and cell morphology [56]. The G protein-coupled receptors (GPCRs) are a family of transmembrane receptors that are relevant to mediate the detection and transduction of signals in response to different environmental factors, and their presence is evolutionarily conserved in practically all organisms [57]. The main objective of any organism in a constantly changing environment is adaptation to the availability of nutrients and stress, so the detection of these external variables is strictly necessary to adapt the morphology, metabolism, and activation of sexual reproduction and the virulence of fungi [55]. In this section, we will discuss the main key pathways in fungal growth and the activation of dimorphism, with emphasis on the stages of these pathways that coordinate the transcriptional regulators directly involved in the change in fungal morphology.

#### 2.3.1. Two-Component Regulatory Systems

**Figure 1:**
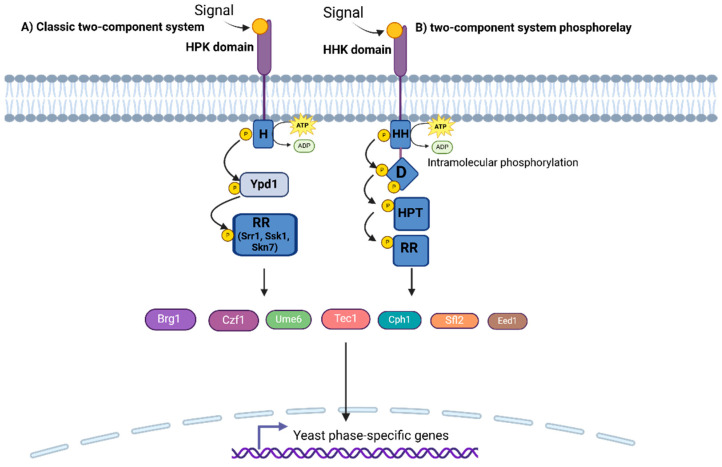
Two-component regulatory system pathway in fungi. (**A**) The classic two-component system based on *C. albicans*, where HPK refers to membrane-bound histidine protein kinase, H is the histidine residue (H box), and RR corresponds to response regulators. (**B**) This is a variation of the two-component system phosphorelay present in some fungi (*H. capsulatum*, *B. dermatiditis*, among others), where HHK refers to hybrid histidine kinase transmembrane receptor, HH and D are the catalytic domains of HHK, HPT is a histidine phosphotransferase, and RR is also a response regulator. Details of the operation of this pathway can be found in the main text. Figure created with Biorender.com.

These signal transduction pathways, strongly conserved in plants and microorganisms, are based on phosphorylation cascades between histidine kinases that end in the phosphorylation of a canonical aspartate residue in the response regulatory protein [58,59]. The two-component systems govern the dimorphism in several pathogenic fungi, such as *C. albicans*, *P. brasiliensis*, *Coccidioides immitis*, *Sporothrix schenckii*, *C. neoformans*, *Blastomyces dermatitidis*, *H. capsulatum*, and *Penicillium marneffei* [28,59,60,61,62]. In addition, these pathways have been associated with morphogenesis control, the response to oxidative and osmotic stress, quorum sensing, and virulence, at least in *C. albicans* [59]. A classic two-component signaling system consists of a membrane-bound histidine protein kinase (HPK) and a response regulator (RR) protein, hence the name “two-component” [59]. The HPK is a dimer composed of two subunits, each of which has an ATP-binding domain, a dimerization domain, and a kinase domain (containing the phosphorylation site, shown in Figure 1, Panel A). Once the HPK input domain is stimulated by an external signal, the dimerization domain of one subunit approaches the kinase domain of the other subunit to promote phosphorylation (Figure 1, Panel A). The degree of HPK phosphorylation affects the level of RR phosphorylation [59]. In this respect, many HPKs could regulate one RR, or an HPK could regulate multiple RRs [59]. The HPKs are distributed and closely conserved in fungi that present the two-component system as a signal transduction pathway [60]. On the other hand, an RR consists of a receptor module and an output domain, where the receptor module regulates the activity of the output domain through the phosphorylation of aspartate residues. In *C. albicans*, three RRs, namely *SSK1*, *SKN7*, and *SRR1*, have been identified [63,64]. Ssk1 and Skn7 play a notable role in morphogenesis and the response to oxidative stress, while Srr1 has implications in hyphal development, and its loss makes *C. albicans* cells sensitive to osmotic and oxidative stresses [63]. The domain or output structure of a two-component regulatory pathway can be a transcription factor that, in turn, controls gene expression or influences the regulation of target protein activity [59].

The two-component system is a multi-step phosphate transduction system in eukaryotes. However, in fungi, it occurs with slight variations. *C. albicans* contains three HPK genes (*SLN1*, *NIK1*, and *HK1*), and there are seven in *C. neoformans* [65,66]. The action mechanism consists of the donation of a phosphate from ATP to phosphorylate a conserved histidine residue (H box) after HPK perceives the stimulant signal (Figure 1, Panel A). The phosphate is then transferred to the aspartate residue of the same HPK receptor domain, followed by transfer to the aspartate residue of the RR receptor domain via the histidine residue of the intermediate transfer protein, Ypd1 (Figure 1, Panel A) [64]. In total, four phosphorylation events occur in an ordered sequence, forming a four-step phosphate transfer system [59,65]. The relevance of Ypd1 is its essentiality for the viability of eukaryotic microorganisms, such as *C. neoformans*, and its interaction with the response regulators Ssk1 and Skn7 has been demonstrated [67]. However, in *C. albicans*, *YPD1* is not essential for viability, and its role seems to be linked to the activation of two signal transduction pathways: the Hog1 mitogen-activated protein kinase (MAPK) pathway, as well as Ssk1 and Skn7 (Figure 1, Panel A), which belong to the two-component pathway [64]. The three *C. albicans* genes seem to have a role in filamentation due to the loss of *NIK1* bringing a severe defect in hyphal formation even when the *nik1*Δ mutant was grown in a culture medium supplemented with serum, an inducer of hyphal development [66]. In general, the loss of *SLN1* and *HK1* also affected hyphal formation compared to the wild-type strain. However, the most striking effect was observed in the *hk1*Δ mutant, where the hyphal formation was abolished [66]. Downstream of the pathway, Hog1 and Skn7 sense the Sln1 signal and regulate later gene expression [68]. The RR Skn7 is a specific and highly conserved transcription factor in ascomycetes and basidiomycetes, and, as we mentioned above, it is required for *C. albicans* hyphal morphogenesis, since it was reported that the loss of *SKN7* has an impact on the hyphal formation of *C. albicans* in solid, but not liquid, media, suggesting that Skn7 plays a highly specific role in surface-induced morphogenesis [58]. In addition, it has also been shown that *SKN7* overexpression induces the formation of hyphae regardless of their environment [69]. Skn7 is a positive regulator of the expression of other transcription factors that positively modulate morphogenesis, such as Brg1, Cph1, Czf1, Eed1, Sfl2, Tec1, and Ume6 [58]. This regulatory network seems to be associated with hyphal differentiation because the formation of biofilms is closely linked to hyphal growth, and some of these transcription factors are responsible for this phenomenon [58].

A variant of the classical two-component system is the so-called phosphorelay, which begins with a hybrid kinase, an alternative form that contains a receptor domain fused to its C-terminal end (Figure 1, Panel B). The hybrid kinase autophosphorylates and performs intramolecular phosphorylation of the receptor domain, then, the phosphoryl group is transported to a histidine phosphotransferase (HPT), and this transfers it to a terminal RR to effect an output (Figure 1, Panel B) [70]. *DRK1* (dimorphism-regulating kinase 1) is a hybrid histidine kinase (HHK) transmembrane receptor highly conserved in some fungi (Figure 1, Panel B). It is characterized by containing the catalytic and receptor domains in the same polypeptide, where the extracellular sensing domain that detects environmental signals is located at the amino-terminal end, and the cytosolic signaling domain is at the C-terminal end [60,62,70]. Accordingly, a report revealed that there was a high percentage of similarity (more than 80%) of the *DRK1* sequences of the species *H. capsulatum*, *B. dermatitidis*, *C. immitis*, *S. schenckii*, *C. neoformans*, *C. albicans*, and *P. brasiliensis* [60]. In *B. dermatitidis* and *H. capsulatum*, *DRK1* acts as a global regulator of dimorphism, and its activity is required for the transition from hypha to yeast [61]. RNA silencing of *B. dermatitidis DRK1* resulted in the arrest of the fungus in hypha morphology even under conditions that normally induce its transition to the yeast form and decreased the transcription of *BYS1*, a yeast phase-specific gene [61]. A similar gene regulation downstream of the pathway occurs in *H. capsulatum*, where yeast phase-specific genes such as *CBP1*, *AGS1*, and *yps-3* are affected by the lack of *DRK1* [61]. In *S. schenckii*, *DRK1* lacks transmembrane domains, but it has PAS and GAF domains belonging to the families of cytoplasmic sensor domains located at the amino-terminal end [61]. The silencing of *S. schenckii DRK1* caused growth defects in the mutants, but no alterations in dimorphism were obtained because the mutants achieved the transition from hypha to yeast, although with notable changes in yeast morphology [62]. The only correlation of Ss*DRK1* with dimorphism is that its silencing caused a decrease in the expression of *Ste20*, a gene related to morphogenesis when Ss*DRK1*-i yeasts were compared to the wild-type strain [62]. The inhibition of *P. brasiliensis* Drk1 with iprodione only delayed the change from hypha to yeast without causing toxicity or inhibiting fungal growth. However, the inhibitor effect transiently maintained Drk1 activity, but no overwhelming effect on arrest was obtained in any cell morphology [60]. Unlike previously described dimorphic fungi, *P. marneffei* has two genes, *DRKA* and *SLNA*, which encode histidine kinases that conform to its two-component system, and it was observed that the non-lethal deletion of *DRKA* and *SLNA* reduced hyphal formation, changed the number of septa, and resulted in abnormal chitin deposits in the cell wall of the hyphae, and it showed a higher rate of phagocytosis [28]. Current antifungal drugs are becoming ineffective against mycoses over time, and new alternatives are continually required to ensure an adequate cure. However, the development of these drugs has been impeded by the structural and functional similarity of mammalian and fungal cells, which has potentially significant harmful consequences if the drugs do not discriminate between host and pathogen cells. Interestingly, hybrid histidine kinases, such as the classic two-component system of fungi in general, are not elements shared with human cells or those of other mammals, which makes them more suitable candidates for therapeutic targets [71,72], hence the importance of continuing the study of the two-component signal transduction pathway in clinically relevant fungi.

#### 2.3.2. Calcium/Calcineurin Pathway

Adaptation to different environmental conditions, which includes the response to various cations, is important for the survival and proliferation of microorganisms. Cells have intracellular signaling pathways, which identify these stimuli and can implement responses to counteract stress conditions [73]. Calcium (Ca^2+^) signaling is preserved in eukaryotes and fulfills several functions, such as the detection of environmental stimuli, the transmission of extracellular signals to the nucleus to modulate the expression of some genes, morphological regulation, responses to abiotic and biotic stress, and defense against pathogenicity and virulence [74,75]. When changes in cytosolic Ca^2+^ levels occur, several detecting proteins, such as calmodulin and calcineurin, can be activated, leading to the induction of different downstream signal transduction pathways [76,77].

**Figure 2:**
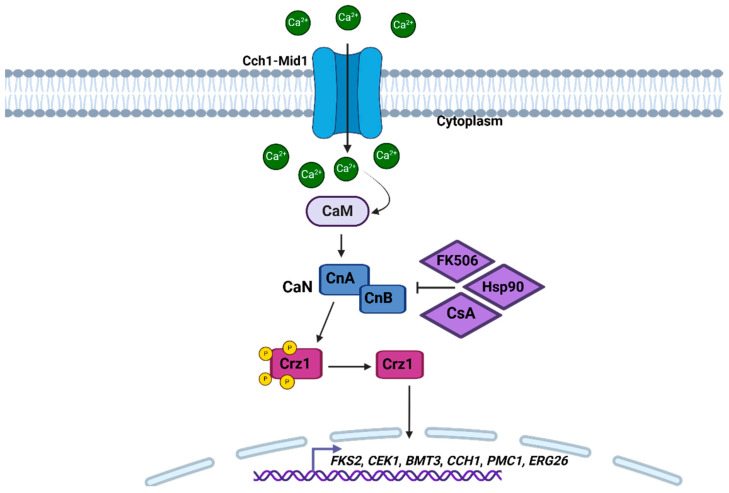
Calcium/calcineurin pathway based on *C. albicans*. The activation of this cascade occurs when the extracellular calcium stimulus is detected by the Cch1-Mid1 channel, which is activated and begins to introduce Ca^2+^ into the cell. Ca^2+^ accumulates in the cytoplasm, and CaM is activated when the Ca^2+^ concentration increases. Subsequently, CaN is activated by CaM, and then, Crz1 is dephosphorylated by CaN. The suggested inhibitors, at least in *C. albicans*, for CaN are FK506, Hsp90, and CsA [88,89,90]. Once the transcription factor Crz1 is active, it alters the transcription of the genes *FKS2*, *CEK1*, *BMT3*, *CCH1*, *PMC1*, and *ERG26*. Figure created with Biorender.com.

Calcineurin (CaN) is known as a heterodimer that has two subunits, the catalytic subunit A (CnA) and the regulatory subunit B (CnB) (Figure 2). Although it is well preserved in the different types of eukaryotes, CaN plays diverse and different roles in organisms [77,78,79,80]. In the pathogenic species *C. neoformans* and *C. albicans*, calcineurin regulates growth at alkaline pH and high temperatures. In addition, it is also responsible for regulating dimorphism, mating, and virulence. In filamentous fungi, it regulates hyphae growth, adaptation to stress, and cell wall integrity [77,81,82,83]. In *H. capsulatum*, this pathway is essential for the regulation of several cellular functions, especially those related to adaptation to stress conditions, contributing to fungal survival at high temperatures, and allowing adaptation to the host milieu [8,84].

In *C. albicans*, Ca^2+^ homeostasis plays an important role in survival and pathogenicity. Under stimulating conditions, the Cch1-Mid1 calcium channel of the plasma membrane is activated, contributing to the entry of Ca^2+^ into the cells (Figure 2) [85]. The increase in Ca^2+^ levels is subsequently detected by calmodulin (CaM) and then activates CaN, which in turn dephosphorizes Crz1 (Figure 2), causing the expression of genes downstream that are dependent on Ca^2+^-CaN, including *FKS2*, *CEK1*, *BMT3*, *CCH1*, *PMC1*, and *ERG26* [86,87]. In *C. albicans*, the study of these genes has been of great interest, because it has been shown that mutant strains in these genes have sensitivity to different antifungal drugs. In addition, several compounds directed to the proteins of this signaling pathway, such as CsA inhibitors, FK506, and Hsp90 (Figure 2), exhibit antifungal activity alone or in combination with other antifungal drugs [85,88,89,90]. It has been reported that changes in *MID1*, *CCH1*, *YVC1*, and *SPF1*, which are involved in this pathway, bring defects in virulence, sensitivity to drugs, such as fluconazole and terbinafine, and changes in hyphal development. In *C. albicans*, it has been shown that *PMC1* has an important role in morphological transition, biofilm formation, and virulence [91,92,93,94,95]. The CaN pathway plays a crucial role in dimorphism, allowing *C. albicans* to respond to environmental signals such as high temperature and pH, which are factors that favor hypha formation [82,96]. CaN-deficient mutants (*cna1*Δ or *cnb1*Δ) had attenuated virulence; however, neither hyphal formation nor growth of the mutants at 37 °C were affected by the loss of CaN [86]. CaN also regulates the ability of *C. albicans* to survive stressful conditions found within the host, such as osmotic stress and cell wall damage. These factors are important for the survival of the fungus during infection. CaN activates genes that reinforce the cell wall integrity, allowing the fungus to resist the host’s immune defenses and the attack by antifungal agents [85,97,98].

In *C. neoformans*, CaN is important for survival at high temperatures, and it is known that, in this fungus, CaN regulates genes that are essential for the response to oxidative stress and osmotic stress and cell wall integrity. The survival of *C. neoformans* at a temperature of 37 °C favors its ability to proliferate in the host’s internal environment. Mutants that lack the genes involved in this pathway are unable to survive at physiological temperatures and show changes in virulence in different infection models [99,100]. In *A. fumigatus*, mutations in *cnaA* and *cnaB* cause defects in germination, the morphology of the hyphae, and the formation of the septa. In addition, it is thought that these mutations are related to changes in the cell wall [101,102,103]. It is known that in this species, the Ca^2+^-CaN pathway regulates the polarized growth of the hyphae, which is essential for colonization and invasion in the host tissues. Unlike other species, in *A. fumigatus*, growth is carried out through the extension of apical hyphae, which require effective machinery for transporting and assembling components of the cell wall. The CaN can regulate this process, since it controls the dynamics of the cytoskeleton, thus facilitating directional growth [103]. In *S. schenckii* and *P. brasiliensis*, the activation of this pathway is critical in the dimorphic transition from hypha to yeast and facilitates the survival of the fungus within the host. In addition to its role in dimorphism, the Ca^2+^-CaN pathway regulates mechanisms that allow the survival of these species in stress conditions, such as oxidative and osmotic stress, and promotes the integrity of the cell wall [104,105].

Despite the important role that Ca^2+^ and CaN play in fungal pathogenicity, their exploitation as a therapeutic target presents significant challenges due to their presence in human cells, where they regulate critical immune functions. However, efforts to develop specific inhibitors for fungal CaN that do not interfere with the human version of the enzyme are an active area of research. Combining CaN inhibitors with conventional antifungals, such as those that affect cell wall synthesis, could offer a synergistic strategy to treat resistant fungal infections. In the future, a deeper understanding of the molecular interactions of CaN with other signaling pathways in fungi could open new avenues for the development of more specific and effective therapies against clinically important fungi [79,101,103].

Finally, the Ca^2+^-CaN pathway also interacts with other signaling pathways that regulate morphogenesis in different fungal species, such as the cAMP/PKA and MAPK pathways, which will be described below. These interactions ensure that the morphological transition and response are effectively coordinated.

Our search did not find any information on this pathway in *H. capsulatum.* So, calcium homeostasis and the role of the CaN pathway are currently areas of opportunity in the study of *H. capsulatum* biology.

#### 2.3.3. Mitogen-Activated Protein Kinase (MAPK)

In eukaryotic organisms, the family of serine/threonine protein kinases, known as mitogen-activated protein kinases (MAPKs), are involved in the transduction of a wide variety of extracellular signals and the regulation of different developmental processes [106]. Sequential activation of the MAPK cascade results in the activation of transcription factors and the expression of specific gene sets in response to environmental stimuli [106]. In addition, this pathway responds to mating pheromones, maintenance of cell wall integrity, changes in osmolarity, and nutrient sensing [107].

**Figure 3:**
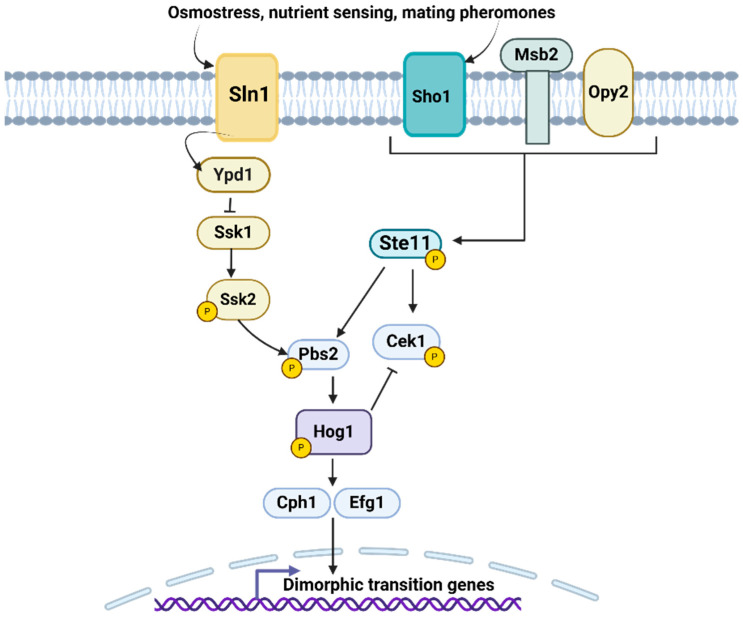
Mitogen-activated protein kinase pathway based on *C. albicans*. The dimorphism-associated environmental signals found in the host that promote the activation of this pathway are nutritional signals, nitrogen deprivation, oxygen availability, serum, and pH. The signal is transferred to the central branch of MAPK, where the Sln1 receptor receives and transmits the signal by sequential phosphorylation of Ssk2 (MAPKKK) and Pbs2 (MAPKK) to the MAP kinase Hog1. The Sho1 branch, in synergy with Msb2 and Opy2, detects and transmits external signals to the Cek1-mediated pathway, which is closely associated with the HOG pathway. Activated Hog1 is then translocated to the nucleus, where it regulates the expression of target genes associated with adaptation to its new environment. The main transcription factors associated with this pathway and involved in dimorphism are Cph1 and Efg1. The figure is based on [109] and created with Biorender.com.

In *C. albicans*, the MAPK pathway is known to be essential for adaptation to different environmental conditions and virulence. In addition, it is involved in many cellular processes such as stress response, morphogenesis, biofilm formation, and antifungal resistance [106,108]. In *C. albicans*, the HOG (high osmolarity glycerol) pathway acts in the adaptation to high osmolarity environments, although this pathway can also be activated by oxidative stress [109]. The HOG pathway of *C. albicans* is activated by two different branches, SLN1, which is based on a two-component system whose proteins are Sln1, Ypd1, and Ssk1, which activates Ssk2 (Figure 3) [109]. The *C. albicans* SHO1 branch is not involved in the activation of Hog1 but in the activation of Cek1. The SHO1 branch is made up of the transmembrane protein Sho1 that, along with Msb2 and Opy2, detects and transmits external signals to the Cek1-mediated pathway, which is intrinsically connected to the HOG pathway. Once Hog1 is activated, it can translocate to the nucleus to regulate the expression of target genes [109].

Signaling through the MAPK pathway activates different transcription factors, such as Cph1 and Efg1, which are responsible for promoting the transition from the yeast to the filamentous form (Figure 3). Mutations in these genes disrupted the development of hyphae or pseudohyphae in response to many stimuli [106,110,111]. The *CEK1* pathway is also one of the key components of this regulation, by activating Cph1. The *C. albicans CEK1* deletion showed defects in hyphal formation in certain solid media, such as SLAD medium, where the nitrogen source is limited [112,113]. *HOG1* also responds to other stress conditions and participates in two important morphogenetic moments of *C. albicans*, filamentation and chlamydoconidia formation [108]. It has been shown that *HOG1* is activated in response to changes in osmotic potential, such as exposure to hypertonic solutions or changes in salt concentration. Such activation allows *C. albicans* to adjust its metabolism and cell structure to cope with adverse conditions. This metabolic adaptation allows the fungus to survive during the transition from the yeast to the filamentous form. *HOG1* works together with other genes, such as *CPH1*, to regulate dimorphism and interacts in different signaling pathways that regulate the morphological transition [108,114,115]. Mutants in *C. albicans HOG1* showed morphological alterations, as a result of a failure to complete the final stages of cytokinesis, with parallel defects in the budding pattern [116]. In addition, these mutants have different colony morphologies compared to the parental strain in some media that promote morphological transition [116].

The *CEK1* and *HOG1* pathways also play an important role in *A. fumigatus*. Activation of *CEK1* is necessary for hyphal formation and conidial production in response to environmental changes. The transition from filamentous to more compact morphologies favors the colonization of host tissues and biofilm formation [117,118]. On the other hand, the *HOG1*-mediated pathway is involved in the stress response, especially under conditions of high osmolarity. These changes allow the fungus to adapt and survive in unfavorable environments. *HOG1* is also related to other signaling pathways that regulate morphogenesis, for example, interacting with *CEK1* [118]. *C. neoformans* has different MAPKs, including Cpk1, Hog1, and Mpk1. However, these are mainly involved in cell wall integrity and maintenance [119]. In *P. brasiliensis* and *S. schenckii*, the activation of MAPK pathways is associated with the transition between yeast-like cells and filamentous forms. In particular, the MAPK pathway appears to be crucial for the transition to the filamentous form in response to environmental factors, such as temperature, pH, and nutrient availability [106,120]. In *H. capsulatum*, there are opposing regulatory pathways that control dimorphism in response to temperature, and the Msb2 regulon that is part of the MAPK pathway has implications for the regulation of yeast-specific genes. During the yeast-to-hyphal transition, in which cells transition from 37 °C to ambient temperatures, about 1100 genes typical of the yeast phase reduce their expression [121]. However, in *msb2* mutants of *H. capsulatum* that were in yeast morphology, 40 of these genes continued to be inappropriately expressed. This group of 40 genes mainly includes direct transcriptional targets of Ryp proteins, indicating that these proteins are still present in the *msb2* mutant even at room temperature. Previous studies showed that, although Ryp protein levels decrease markedly in wild-type cells at room temperature, in the *msb2* mutant, these proteins remain abundant. These results suggest that the Msb2 pathway is crucial in preventing the accumulation of Ryp proteins at low temperatures. However, at 37 °C, the transcription factor Ryp3 binds to the *MSB2* promoter region and reduces its expression, implying that the circuit of Ryp proteins counteracts the Msb2 pathway at high temperatures [29]. Thus, *Histoplasma* manages to switch between states in response to temperature through these opposing regulatory pathways [29].

The MAPK pathway is a key component in the biology of pathogenic fungi and represents an important target in the development of new antifungal drugs. The modulation of this pathway may offer innovative strategies to combat fungal infections, tackle drug resistance, and improve the effectiveness of existing therapies.

#### 2.3.4. Cyclic AMP-Dependent Protein Kinase A

**Figure 4:**
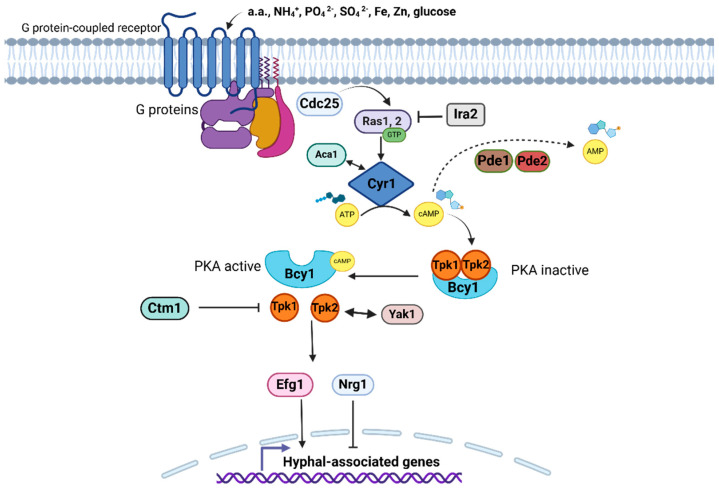
Schematic representation of the cAMP/PKA signaling pathway in pathogenic fungi. This pathway responds to nutrient availability, particularly carbon and nitrogen sources, through G protein-coupled receptors (GPCRs) in the cell membrane. GPCR activation stimulates cyclic AMP (cAMP) production by adenylate cyclase (Cyr1), which in turn activates protein kinase A (PKA). PKA phosphorylates various effector proteins, including transcription factors, structural regulators, and enzymes, promoting key physiological responses such as morphological transition and the expression of virulence factors in fungal pathogens. In *C. albicans*, Ras1 and Ras2 regulate the activation of the adenylate cyclase Cyr1. The phosphodiesterase Pde1/Pde2 modulates cAMP levels, controlling PKA activity. In *C. albicans*, PKA activation regulates factors such as Efg1 and Nrg1, modulating the yeast-to-hyphal transition. Figure created with Biorender.com.

The cyclic AMP(cAMP)-dependent protein kinase A (PKA) pathway is involved in the perception and adaptation of fungi to the inhospitable environmental changes that occur during their life cycle, particularly in adaptation to nutrient accessibility [122]. The cAMP/PKA pathway is activated in response to nutrient availability in the environment, especially carbon and nitrogen sources, such as glucose and amino acids, which are sensed by G protein-coupled receptors (GPCRs) located on the cell surface (Figure 4) [122]. The detected signal results in the activation of guanine nucleotide-binding proteins (G proteins) that induce an increase in the production of intracellular cAMP mediated by the adenylate cyclase enzyme [122]. The high cAMP concentration, known as the second messenger, activates cAMP-dependent protein kinase A, which subsequently phosphorylates target proteins located downstream (Figure 4). The identity of these proteins in the final part of the pathway includes transcription factors, structural proteins, or enzymes that carry out many responses due to the activation of this pathway [122]. The morphological transition and the expression of virulence factors are among the responses derived from the activation of this signaling pathway in medically relevant fungal pathogens [122,123]. This pathway has been identified in *C. albicans*, *C. neoformans*, *P. brasiliensis*, *M. rouxii*, and *P. marneffei* [124,125,126,127,128].

In *C. albicans*, there are two Ras GTPases, namely Ras1 and Ras2, a single adenylate cyclase, Cyr1 (also named as Cdc35), the low- and high-affinity cyclic nucleotide phosphodiesterases, Pde1 and Pde2, respectively, and protein kinase A conformed of regulatory and catalytic subunits (Figure 4) [129]. Cdc25 is a guanine nucleotide exchange activator of Ras1 and Ras2, while Ira2 is its inactivator [129,130]. Similarly, in *C. neoformans*, there are seven GPCRs that detect glucose (Ste3/Cpr, Cpr2, and Gpr1–5), and several G proteins acting downstream of GPCRs have been identified. These are three subunits, Gα (Gpa1/3), Gpb1, Gib2, and Gpg1/2, where Gpa1 and Gib2 specifically participate in the cAMP/PKA pathway, Cac1 is the only adenylate cyclase, Aca1 induces Cac1 activity, Ras1/2 are Ras GTPases, and the PKA consists of two catalytic subunits and one regulatory subunit [124]. In *C. albicans*, Ras1 plasma membrane localization is determined by C-terminal cysteines, although it is not clear whether it is anchored to the membrane or is in the cytoplasm, but it is relevant to hyphal development [131]. Otherwise, Ras2 is special because it has low sequence similarity to other Ras proteins, but maintains the common CCIIT tag for membrane anchoring, suggesting that it is also associated with the plasma membrane [132]. Notably, Ras2 seems to have a lesser impact on morphogenesis; however, it acts synergistically with Ras1, and both proteins have an antagonistic role in controlling the concentration of cAMP since mutants lacking Ras1/2 present a cell morphology affected by both a cAMP-dependent and cAMP-independent mechanisms [129,130,132].

The activation of this pathway is mediated by Cdc25, which activates Ras1/2. In *C. albicans*, the loss of Cdc25 affected the growth rate in both liquid and solid media, and the cytotoxic effect against oral epithelial cells, despite the fact these cells are not capable of filamentation [130]. In this regard, filamentation is a *Candida* evasion strategy against the macrophage-mediated immune response [133], and, unexpectedly, it was observed that the hypofilamentous *cdc25*∆ null mutant had an even higher survival rate than the wild-type strain, suggesting that other factors in addition to morphogenesis participate in the immune evasion mechanism [130]. The *C. neoformans* pathway activation consists of the coordination between the Gpa1 and the adenylyl cyclase-associated protein Aca1, and both induce the Cac1 activity. The role of Aca1 is relevant for activation, and its elimination leads to problems in mating, capsule formation, and melanin synthesis. However, these phenotypes are restored with the external addition of cAMP [124,134]. An alternative route for Cac1 activation is the transformation of CO_2_ to bicarbonate, a reaction catalyzed by the carbonic anhydrase Can2. Therefore, the cAMP/PKA pathway can be alternatively triggered by host stimuli [124,135]. Both Cyr1 and Cac1 synthesize cAMP through cyclization of adenosine triphosphate (ATP), and this second messenger binds to the PKA regulatory subunit for its activation [124,130]. Similarly, the transient increase in Cyr1 is associated with the onset of dimorphic change in *P. brasiliensis*, suggesting that this pathway is actively involved in controlling dimorphic change [125].

The cAMP level is regulated by phosphodiesterases, and the *C. neoformans* genome contains *PDE1* and *PDE2* genes with such predicted activity. However, only Pde1 actively participates in the control of cAMP concentration, whereas in *C. albicans*, both Pde1 (with low-affinity) and Pde2 (with high-affinity) phosphodiesterases participate in cAMP regulation and are associated with hyphal growth (Figure 4) [136,137]. *C. neoformans PKA1* and *PKA2* encode for the catalytic subunits of PKA, whereas *PKR1* encodes for its regulatory subunit, and the whole holoenzyme is involved in melanin synthesis and capsule formation [124]. On the other hand, *C. albicans PKA* is integrated by catalytic subunits Tpk1 and Tpk2 as well as the regulatory subunit Bcy1, and the protein complex acts on transcription factors associated with filamentation (Figure 4) [138]. Recently, it has been proposed that a dual-specificity tyrosine phosphorylation-regulated kinase, Yak1, acts downstream or in synergy with PKA to regulate filamentation in *C. albicans* since Yak1 is required for the yeast-to-hyphae transition (Figure 4) [138]. In contrast, the methyltransferase Ctm1 is a crucial regulator in hyphal morphogenesis in *C. albicans*, as unmethylated Ctm1 binds to Tpk1 and Tpk2, inhibiting their kinase activity, which suppresses downstream regulation of the pathway by preventing hyphal development [127].

Once the PKA holoenzyme is activated, its catalytic subunits activate several effector proteins (this encompasses transcription factors) downstream of the pathway through phosphorylation [130]. A transcription factor highly associated with this pathway is Efg1 (Figure 4), which regulates the expression of genes involved in morphogenesis, the white–opaque phenotypic change, among other traits related to *C. albicans* virulence [139,140]. *C. albicans* Efg1 is homologous to *Saccharomyces cerevisiae* Sok2 and Pdh1, which are other transcription factors with functions in pseudohyphal growth, all of them belonging to the APSES family (Asm1, Phd1, Sok2, Efg1, and StuA) of transcriptional regulators (Figure 4) [141]. *C. albicans* Efg1 has a dual negative and positive impact on hyphal development based on environmental stimulus and signaling cascades, such as cAMP/PKA, because it directly regulates hyphal-associated gene expression [141]. Nrg1 is another transcription factor whose decreased expression depends on the cAMP/PKA pathway, and it plays an important role in repressing hyphal formation [142]. *C. neoformans* Nrg 1 is distantly related to the *C. albicans* ortholog, sharing only PKA consensus sequence similarity for its phosphorylation, but it is positively involved in cAMP-associated phenotypes, such as capsule formation and mating, but not with melanin synthesis [143,144]. The *C. neoformans* genes downstream of PKA are mostly related to environmental stress responses, such as heat shock proteins acting in response to stress and susceptibility to azole drugs, but not with hyphae formation [143,144]. In conclusion, the cAMP/PKA signaling pathway of *C. albicans* is associated with the control of the yeast-to-hyphal phenotypic change; however, in *C. neoformans*, this pathway contributes more to maintaining pathogenesis against stressful environmental factors and is not involved in hypha formation [127,145].

#### 2.3.5. Pal/Rim Pathway

**Figure 5:**
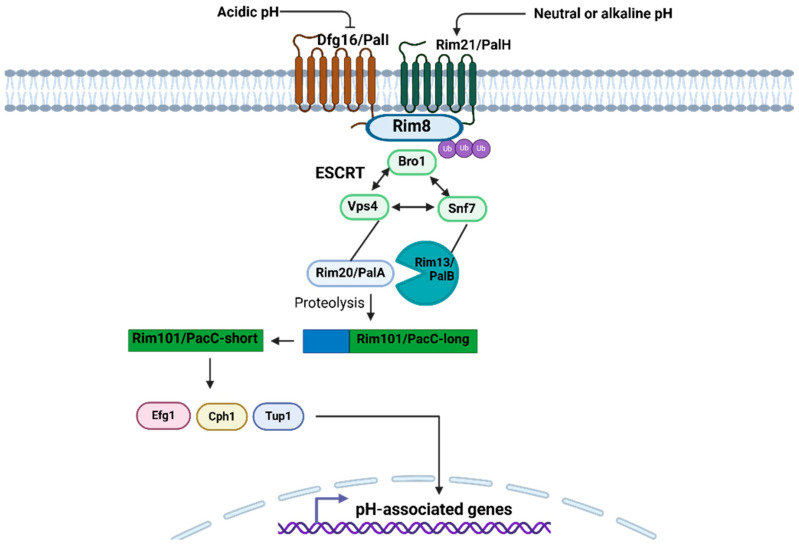
Schematic representation of the Rim/Pal signaling pathway in fungi. This pathway regulates fungal adaptation to environmental pH, playing a key role in dimorphism and virulence. In response to alkaline pH, membrane-associated sensors (Rim21/PalH and Dfg16/PalI) activate a proteolytic cascade, leading to the cleavage and activation of the transcription factor Rim101/PacC. The active form translocates to the nucleus, modulating gene expression to promote filamentation in *C. albicans* or other adaptive responses in different fungi. In *C. neoformans*, Rim101 is also linked to the cAMP/PKA pathway, integrating additional environmental signals such as iron availability. ESCRT complexes (I–III) and endosomal components (Snf7, Vps4, Bro1) participate in the processing of Rim101/PacC. Created with Biorender.com.

Environmental pH and its variations have a significant influence on microorganisms. Therefore, they have to sense and respond to these stimuli to maintain survival [146]. Different fungal species, as well as other microorganisms, share signaling pathways in response to environmental pH, and one of them acts in response to an alkaline pH signal, namely Pal/Rim, which is very important for the fungal pathogenesis and secretion of various low-molecular-mass molecules into the extracellular medium [146,147]. The Rim/Pal pathway involves the activation of a zinc finger transcription factor, PacC in filamentous fungi, or Rim101 in yeast-like fungi, through a proteolytic cascade [147]. This master regulator, in turn, positively or negatively regulates the transcription of other genes involved in many physiological tasks, including dimorphism [147,148]. The general components of this pathway are conserved among filamentous and yeast-like fungi, such as *C. neoformans* and *C. albicans* [148,149,150]. The H. capsulatum Rim/Pal elements are unknown, so research is needed to elucidate their relevance in the dimorphism of this organism. However, among fungal species, there are notable particularities, especially in the transcriptional regulation downstream of this pathway [146]. This transcriptional specificity probably allows fungi a distinctive adaptation to species-specific environmental niches [146]. For example, pathogenic fungi of humans and other mammals can survive the unfavorable conditions of the host microenvironments, where the pH can range from 2 to 10 because of signaling pathways that are essential for proper cellular functioning [146]. In human commensal fungi that colonize mucosal anatomical cavities, such as the gastrointestinal, oral–pharyngeal, and genitourinary tracts, and that are also opportunistic, with the ability to spread until reaching any organ, a challenge is implied to recognize extracellular pH changes to maintain cellular homeostasis; therefore, it is not surprising that some of these fungi, such as *C. albicans*, have the Rim101 pathway [146,148,151], where the acidic pH favors the growth of its cells in the form of yeast, and, on the other hand, the neutral-alkaline pH promotes hypha formation [152]. However, the control of dimorphism depending on pH can occur oppositely, for example, in *Trichosporon cutaneum*, the acidic pH drives filamentation, while the neutral-basic pH induces the growth of cells in yeast-like shape [153]. Extracellular pH recognition is mediated by the plasma membrane-associated receptor proteins Rim21/PalH and Dfg16/PalI (Figure 5), where alkaline pH activates the Rim101/PacC pathway, and, in contrast, the acidic pH does not favor proteolysis-mediated activation of Rim101/PacC or is activated differently [151,154] (Figure 5). The activation of Rim21/PalH and Dfg16/PalI by neutral-alkaline pH promotes the ubiquitination of the Rim21-associated protein Rim8, which contributes to endocytosis [151] (Figure 5). *C. neoformans* Rim101 is intimately connected to the cAMP/PKA signal transduction cascade, where Rim101 activation requires phosphorylation by PKA. The relationship of these two pathways seems to assign Rim101 a flexible role in the upstream signals that allow its activation. *C. neoformans* Rim101 does not seem to be only a pH-responsive factor but can respond to multiple host stimuli through the cAMP/PKA pathway, including other environmental signals, such as low iron levels or tissue culture medium [155].

Subsequently, Snf7 is oligomerized on the endosome surface and joins to Rim13/PalB, a calpain-like protease, and Rim20/PalA, another protease that binds to C-terminal inhibitory domain of the inactive transcription factor Rim101/PacC-long, which is engaged in the endosomal membrane [151,154]. In addition, Rim20 interacts with Vps4, which in turn interacts with Snf7 [154] (Figure 5). Bro1 was later found to interact synergistically with Vps4 and Snf7 in the transport of multivascular bodies (MVBs) to the vacuole [154]. Transport of MVBs to the vacuole needs three protein complexes, termed ESCRT-I-III (Endosomal Sorting Complex Required for Transport) [154]. Snf7 is a cytoplasmatic protein of the ESCRT-III protein complex that is recruited to endosomal membranes; Vps4 is a cytoplasmic AAA-type ATPase that is present in the three ESCRT complexes to recycle components of the pathway; and Bro1 is also a cytoplasmic protein that is associated with MVB and required Vps4 for the dissociation of ESCRT-III [156]. Returning to Rim101/PacC, once Rim13/PalB performs the proteolytic processing on the C-terminal inhibitory domain of Rim101/PacC, its active form is obtained, which is suitable for translocating to the nucleus and regulating gene transcription associated with responses dependent on neutral-alkaline pH [148,151]. The identification of this pathway in *C. albicans* began with a differential expression analysis that identified two genes induced by alkaline pH, namely, PRA1 and PHR1 [148]. The PRA1 (pH-regulated antigen) gene encodes a protein located in the cell wall whose role is involved in alkali-induced filamentation; the PRA1 null mutant had a temperature-dependent defect in the initiation of filamentation, indicating its role in morphogenesis [157]. PHR1 is another pH-associated gene in *C. albicans*, whose expression was induced at alkaline pH and was repressed when pH values were lower than 5.5 [158]. The mutant lacking the two PHR1 alleles showed a defect in its morphology conditioned by pH, since, at alkaline pH, this mutant could not form hyphae or yeasts, as could the parental strain used as a control [158]. RIM101, RIM 20, and RIM8 are components of the only signaling pH-pathway Rim101p/PacC in *C. albicans* [148]. RIM101 encodes a pH-inducible zinc finger transcription factor that is dependent on Rim20 and Rim8 [148]. The operation of this pathway begins when the alkaline pH of the medium induces Rim8 and Rim20 proteolysis of Rim101p-long until it is processed into its Rim101p-short form, ready to carry out a response, that is, the activation (such as PHR1) or repression (e.g., PHR2) of genes that coordinate induced filamentation by alkali [148,159]. Finally, Rim101 activates filamentation by interacting with either the positive regulators Cph1 and Efg1 or the negative regulator Tup1 [148]. Efg1 is required for Rim101-induced filamentation but is not necessary for alkali-induced gene expression, and Tup1 is not regulated during filamentous growth; otherwise, their associated DNA-binding proteins are likely regulated [159]. Thus, in *C. albicans*, Rim101 is a positive regulator of filamentation, as it acts strongly on ECE1, CSA1, CSA2, SAP5, HYR1, HWP1, RBT1, and IHD1 [160]. Unlike *C. albicans*, in *C. neoformans*, the role of Rim101 is related to the formation of the capsule and the fungal growth in various stressful environments, such as low iron concentrations, high salinity, or alkaline pH [150,155].

## 3. Transcriptional Regulation of Fungal Dimorphism

Signals derived from the environment or the host, such as nutrient availability, light, gases, stress, temperature, and pH, mainly activate the change in the morphology of fungal cells, through the signaling pathways previously described above, which ends in the transcription of genes that direct dimorphism [28,56,159]. The ability of fungi to transition from yeast to hypha or hypha to yeast in response to environmental factors is not only important to their pathogenicity, but additionally, this phenomenon is an excellent model for understanding how signaling pathways govern the growth and development of these organisms [159]. The activation of phase-specific genes in either hyphae or yeast contributes to cell cycle deregulation, the establishment of polarity, and cell wall changes [58]. Fungal morphogenesis is under the control of a complex network of transcriptional regulators that act negatively or positively on the expression of phase-specific genes [58]. The negative regulation of hyphal-specific genes (HSGs), as it appears in *C. albicans*, is driven by the transcriptional repressor Tup1, in association with the DNA-binding proteins Nrg1, Rfg1, and Sfl1 in synergy with the corepressor Ssn6 [58,161]. The positive regulation of HSGs is controlled under almost all conditions that induce hyphal formation by the transcription factors Skn7, Efg1, Flo8, and Ndt80, while other transcriptional factors, such as Cph1, Czf1, Tec1, Sfl2, and Rim101, positively regulate HSGs in a condition-specific manner or for each activated signaling pathway [58,68,152]. These transcriptional factors act mainly at the beginning of morphogenesis, and their participation continues during hyphal growth; however, the transcriptional factor Ume6 has a major role in hyphal development [58]. Chromatin remodeling is a phenomenon that is also involved in morphogenesis since it is necessary for the transcriptional activation of face-specific genes. In this sense, it has been shown that Brg1 binds to HSG promoters to counteract the repression mediated by the Nrg1-Tup1 complex, which promotes chromatin remodeling and favors HSG transcription [58,111,161].

In dimorphic fungal pathogens such as *C. albicans*, the role of genes upregulated in the yeast morphotype is fundamental and has been extensively studied, but genes that are silenced in yeast morphology, i.e., mold-specific genes in filamentous fungi that transition to yeast, also play a critical role in dimorphism and have not been described in the same detail.

## 4. Dimorphism Regulators with Therapeutic Potential

Current therapy against fungal infections caused by *Aspergillus*, *Candida*, or *Cryptococcus* species is focused on target cellular components or processes such as ergosterol, sterol 14α-demethylase, β-glucan synthesis, and interference with protein, RNA, or DNA synthesis [162,163]. However, each of these strategies has its drawbacks and limitations, such as high toxicity as well as the latent risk of developing drug resistance [162,164]. For this reason, new therapeutic approaches continue to be sought and proposed, such as drug repositioning, and the use of natural or synthetic compounds modified to target other structural components of the cell wall, the plasma membrane, the regulation of gene expression, virulence factors, and even the signal transduction pathways [162,163]. Considering the drawbacks of conventional antifungal therapy, as well as the relevance of the sanitary control of mycoses, the signaling pathways involved in the dimorphism of pathogenic fungi of clinical interest could have a wide potential for the development of new drugs against some elements that regulate these cascades [162]. The dimorphic regulatory histidine kinase 1, Drk1 (described in detail in the Two-Component Regulatory Systems Section), has been proposed as a key factor for inhibitory analysis in clinically relevant species, such as *S. schenckii*, *P. marneffei*, and *P. brasiliensis* [62,165]. In a study involving fludioxonil, a group III histidine kinase inhibitor (iDrk1), it was suggested that *P. brasiliensis* Drk1 functions in fungal resistance to different cell wall disrupting agents by reducing viability after iDrk1 treatment [166]. In addition, a significantly higher phagocytic index was also observed in iDrk1-treated yeasts than in the control group through phagocytosis assays, suggesting the possible role of PbDrk1 in cell wall modulation, making it a relevant target for further investigation [166]. Taking into consideration these multifaceted peculiarities of Drk1, a study was recently performed using a phage display technique to identify peptides capable of interacting with Drk1 of *P. brasiliensis*, and the effects of these interactions on the association with the *P. brasiliensis* host were examined [162]. The findings of the study were promising, as the peptides were found to be able to inhibit the morphological phase transition of *P. brasiliensis*. Furthermore, a substantial proportion of these peptides did not allow adhesion to pneumocytes. Although these peptides may not have inherent antifungal properties, they potentiate the effects of some antifungal agents. The efficacy of the peptides was also evaluated in the alternative host *Galleria mellonella*, and it was shown to contribute to improved larval survival rates [162]. Due to it being suggested that Drk1 is present only in bacteria and fungi, it is of interest to continue its physiological characterization, but even more important would be to persist with developing new therapeutic proposals directed against Drk1 [164].

The calcium/calcineurin signaling pathway of *C. albicans* has also been proposed as a candidate for the development of inhibitors to control infection by this opportunistic fungus [85]. Specifically, proteins that modulate the intracellular concentration of Ca^2+^ in both *C. albicans* and humans are of interest, since they have a low level of identity at the amino acid sequence level. For example, Cch1 has a sequence identity of 11.2% with its human counterpart. Also, the functional domains III S1, S2, and S3 of *C. albicans* Cch1 share little identity, with 11.1%, 34.8%, and 20.0%, respectively, compared to the domains of human Cch1 [85]. It is relevant to highlight that Mid1 and Yvc1 are fungi-specific proteins of the Ca^2+^-CaN signaling pathway, which gives them the potential to be desirable targets for the development of drugs for the control of candidiasis [85,167]. *C. albicans SPF1* encodes the calcium ATPase, which plays a role in maintaining cellular calcium homeostasis, and *SPF1* deletion results in defects in hyphal development and strongly attenuates *C. albicans* virulence. Furthermore, this mutant was hypersensitive to test antifungal drugs, such as fluconazole, tunicamycin, and hygromycin B [91]. Pmc1p is a Ca^2+^ pump that also plays an important role in calcium homeostasis, as well as having played a role in the morphological transition, biofilm formation, and virulence in a mouse model of disseminated infection. It was shown that the reduced susceptibility of the homozygous *pmc1* mutant to fluconazole was temperature-dependent and, therefore, may be related to a form of azole tolerance [95]. CaN plays a central role in this and other signal transduction pathways; however, in *C. albicans*, it has been reported that the deletion of this gene produces in the mutants a greater susceptibility to fluconazole and a decrease in its virulence during pulmonary infection [97,168]. Therefore, CaN appears to have a favorable potential to be targeted by antifungal drugs that cause its dysfunction. Despite the complete CaN protein of humans and fungi sharing a similarity greater than 40%, some functional domains of fungal CaN have clear differences, which is encouraging for its use in the development of inhibitory compounds that specifically bind to these domains and serve to combat *C. albicans* infections [85]. The transcriptional factor Crz1 and the protein Hsp90 also show promising potential as targets for the development of antifungal drugs, and their possible use value is also due to the low levels of similarity with the human orthologs [85]. The Hsp90 chaperone has promising potential for drug design, as the determination of the crystal structure of the N-terminal domain of *C. albicans* Hsp90 provided relevant information about the notable structural differences between fungal Hsp90 and its mammalian counterpart, despite the high sequence conservation within the nucleotide-binding domain [169].

The signaling involved in pH changes has also been addressed with pharmacological approaches to combat fungal infections caused by *A. fumigatus*, *C. albicans*, and *C. neoformans*, among others; specifically, attention has been paid to the Rim/Pal pathway as a source of targets to propose antifungal strategies [160]. Evidence of this is that the disruption of *VPS28* and *VPS32*, two Vps factors required for Rim pathway activation, increased the susceptibility of *C. albicans* to drugs directly involved with cell wall assembly at the level of chitin synthesis and deposition or the level of the construction of the β-1,3-glucan layer independently of the Rim pathway [170]. Recently, research on the Rim pathway has continued, and it is now known that this entire cascade is associated with tolerance to azole compounds and echinocandins since the elimination of any of its elements brings with it hypersensitivity [171,172].

Within the protein kinase pathways, there are also distinguishable therapeutic targets, such as the cyclic AMP protein kinase A (cAMP-PKA) pathway, where the catalytic subunits Tpk2 and Cyr1 play an important role in the regulation of tolerance to drugs through the regulation of efflux pump expression [172]. The relevance of the entire cAMP-PKA pathway has been studied together with its correlation with the regulation of susceptibility to antifungals in *C. auris*, and it is known that the pathway is closely related to antifungal resistance, such as to amphotericin B, fluconazole, 5-fluorocytosine, caspofungin, and the fungicide fludioxonil [163]. Thus, it has been proposed that the development of inhibitors of the cAMP/PKA pathway could be favorable for the treatment of *C. auris* infection, in combination with some currently available antifungal drugs. Future perspectives are focused on the characterization of possible upstream regulators of Cyr1, such as a G protein-coupled receptor or small or heterotrimeric GTP-binding proteins; the identification of Cyr-independent upstream regulators of PKA; and the clarification of Cyr1- and PKA-dependent downstream signaling networks [163].

## 5. Conclusions

Many of the mechanisms described above support the hypothesis that some key elements of these pathways could be targets to develop new therapeutic approaches against fungal infections. We propose that it is necessary to continue investigating in detail each of the master regulators of dimorphism, especially those that are not shared with the mammalian hosts, that are conserved throughout the fungal kingdom, and that have promising potential as therapeutic targets. Understanding the key master regulators of these pathways in detail is a crucial step, since although there are many proteins involved in these processes with the potential to be inhibited, only those target elements that are highly conserved in pathogenic fungi and specific to the fungi kingdom should be selected. These relevant fungal-specific elements should be characterized from a genetic, biochemical, physiological, and pharmaceutical perspective to achieve greater efficiency in the development of new drugs. Some of the signaling pathways and their mechanisms described here, such as two-component regulatory systems, the calcium/calcineurin pathway, the mitogen-activated protein kinase pathway, the cyclic AMP (cAMP)/protein kinase A pathway, and the Rim/Pal pathway, are very well understood. However, it is possible that there are additional signaling pathways that are also involved in regulating dimorphism and that have not yet been identified. It is also crucial to know precisely the interweaving of the genetic networks that exist with other metabolic pathways that could be shared in humans and other mammals to guarantee the efficacy of the drugs that are designed based on this knowledge.

## Data Availability

Not applicable.
